# Cross-sectional study of self-reported physical activity, eating habits and use of complementary medicine in breast cancer survivors

**DOI:** 10.1186/1471-2407-13-153

**Published:** 2013-03-25

**Authors:** Arnoud J Templeton, Beat Thürlimann, Michael Baumann, Michael Mark, Sarah Stoll, Madeleine Schwizer, Daniel Dietrich, Thomas Ruhstaller

**Affiliations:** 1 Breast Centre, Rorschacherstrasse 95, St. Gallen, 9007, Switzerland; 2 Statistics Department, Swiss Group for Clinical Cancer Research (SAKK), Bern, Switzerland

**Keywords:** Breast cancer, CAM, Eating habit, Interest, Physical activity, Survivor

## Abstract

**Background:**

Besides conventional adjuvant therapies, many breast cancer survivors engage in various activities like exercise, diet and complementary and alternative medicine (CAM) in order to improve their prognosis. Little is known about specific interests and willingness to participate in institutional programs (e.g. exercise classes).

**Methods:**

We conducted a cross-sectional study in patients with early breast cancer assessing current physical activity (PA, e.g. 30 minutes brisk walking), attention to eating habits (“diet”), use of CAM, and interest in learning more about these fields. Patients indicating interest in PA counselling received a voucher for a free instruction by a certified physiotherapist. Data were analysed for factors predictive for engagement in the three fields using a stepwise multivariate logistic approach.

**Results:**

Of 342 consecutive patients, 232 (69%) reported to be physically active more than once per week, 299 (87%) paying special attention to nutrition (in most cases fruits, “balanced diet”, low fat), and 159 (46%) use of CAM (vitamins, special teas, homeopathy, herbal medicine, mistletoe). Factors predictive for PA were use of CAM, higher age, and fewer worries about the future. Swiss nationality at birth, physical activity and higher education were predictive for diet; whereas physical activity, higher education and lower age were predictive for use of CAM. No associations between any of the above variables and breast cancer characteristics were found. Around half of the patients reported interest in receiving more information and willingness to attend special counselling. Of 166 vouchers, only 7 (4%) were eventually utilized.

**Conclusions:**

A high proportion of breast cancer survivors report PA, following a specific diet and use of CAM. There were no disease related factors associated with such pursuits, but an association between patient related factors and these fields was observed suggesting general health awareness in some patients. Around half of the patients were interested in more information and indicated willingness to participate in institutional programs. Impact on disease specific and general health including health economic aspects warrants further research.

## Background

Women with early breast cancer may suffer from toxicities of adjuvant therapies or radiation treatment, may feel fatigued, anxious, or in a low mood state and some become fearful of overexertion and are uncertain of what they can do [1,2]. In daily practice, many patients raise questions about what lifestyle measures they can modify to improve their health status and disease specific prognosis [3].

Physical exercise has been shown to improve self-esteem, fitness and coping with therapy and there is evidence that physical activity is associated with reduced all-cause and breast cancer-specific mortality [4].

Several studies have suggested that dietary interventions may improve relapse-free survival in breast cancer patients with differential effects on hormone-receptor positive and negative disease [5-7] whereas other trials did not show a reduction in breast cancer events or mortality during a 7-year follow-up period [8].

Complementary and alternative medicine (CAM) is a growing field in health care and particularly among breast cancer patients [9-12]. To date, there is no convincing evidence that use of CAM has a major impact on breast cancer outcome [13,14]. Several CAM interventions have reported an association with improved quality of life (QoL), increased coping effectiveness and alleviation of hot flushes [15-18].

Though many patients ask for supportive options there are sparse data on interest of and factors associated with willingness to participate in institutional programs (e.g. exercise classes). The aim of this cross-sectional study was to investigate what activities Swiss patients with early breast cancer are carrying out and what fields they are interested to receive further information about and/or are willing to actively engage in. The characteristics of these patients were also explored.

## Methods

### Study population

Consecutive patients from the Breast Centre St. Gallen (a tertiary referring centre in Eastern Switzerland) were offered study participation. Inclusion criteria were a diagnosis of early breast cancer with no sign of recurrence. All patients provided written informed consent for study participation. Ethical committee approval was received on 11-NOV-2008 (EKSG 08/082/2B) by the *Ethikkommission des Kantons St. Gallen* and the study was conducted in accordance with the Declaration of Helsinki and its subsequent amendments and according to Good Clinical Practice (GCP) guidelines.

### Data collection

All consenting patients were asked to complete a questionnaire (help of the attending nurse was available, if requested) about habits in the fields of physical activity (defined as > 30 minutes brisk walking or equivalent once or more per week), attention to eating habits (“diet”), and use of CAM. Specifically, the questions for eating habits and CAM were “Do you pay special attending to your nutrition?” and “Do you use something from the field of complementary medicine?”, respectively. Both questions could be answered with “yes” or “no” and, in case of “yes” a selection of various modalities was offered including “other” (to be specified) and allowing multiple answers. Patients were also asked if they wished to receive further information and about their willingness to present at a separate occasion for special counselling in their respective fields of interest. Patients indicating interest in more information or willingness to participate in institutional programs (e.g. exercise classes), were offered a free voucher for personal counselling and guided physical training by a certified physiotherapist. Further questions referred to smoking habits, mood, type of health insurance, education, nationality, living situation (alone or with partner, rural or urban), employment situation, and if breast cancer diagnosis had lead to a general change in life. After completion of the questionnaire weight, height and vital signs were recorded and body composition (using Bio Impedance Analyse, BIA 4plus, 83125 Eggstätt, Germany) and peak flow capacity measured. Patient and tumour characteristics including age at diagnosis, tumour stage, pathological grade, hormone-receptor and Her2-status, adjuvant treatment, and concurrent disease were extracted from hospital records. For patients refusing to participate in the study, a non-compliance form was completed documenting the reasons for decline (if given).

### Statistical analyses

Objectives of this study were to evaluate what patients with early breast cancer do and are possibly willing to do beside classical adjuvant treatment and to identify predictors for PA, attention to eating habits and use of CAM. We prospectively hypothesized that patients engaging in any of these fields would have a higher socioeconomic status (i.e. private insurance, higher education), be younger, non-smokers, have a lower body mass index (BMI), and no comorbidities.

In a logistic regression model with a binary response variable (no PA or once per week vs. more than once per week and yes vs. no for attention to eating habits and CAM, respectively), and a binary independent variable (two groups) and assuming that at least 25% of the patients would be in either group, a sample size of n = 333 was necessary to detect a difference of 20% in response rates with a power of 80% at a significance level alpha of 0.05 and allowing correlation between the binary predictor of interest and several covariates (R-squared = 0.25). Each independent variable was first analysed in the univariate setting and then all variables with a p-value < 0.1 analysed together in a multivariate model using a stepwise model search to find the relevant predictors. Sample size estimation was conducted with PASS 2008 (NCSS, LLC, Kaysville, Utah, USA) and analyses were performed with S + 8.2 (TIBCO Software Inc., Somerville, MA, USA).

## Results

Between December 2008 and September 2010 375 patients were asked to participate in the study while attending the Breast Centre of St. Gallen. 28 (8%) patients declined participation and 5 (1%) patients were excluded from the analysis because they did not fulfil the selection criteria (e.g. no history of breast cancer, metastatic disease) or withdrawal of consent. All remaining 342 (91%) patients completed the questionnaire. Patients’ characteristics, concurrent and prior therapies are presented in Table 1. Median age was 61 years, most patients were Swiss citizens (78%), and median time since diagnosis of breast cancer was 3.1 years. Patients had stage I, IIA, IIB, and IIIA/B in 29%, 35%, 20%, and 16%, respectively, with around 75% endocrine sensitive and 16% Her2 positive disease. Concurrent and prior treatment was typical for patients with early breast cancer.

**Table 1:** Patients’ characteristics and treatment (n = 342)

	**n**		**%**
Age; median (range), years		61 (29–94)	
Time since first diagnosis; median (range), years		3.1 (0.1 - 35)	
Postmenopausal	298		87
Mood; median (range), VAS (0 = unhappy, 10 = happy)		7.2 (0.8 - 10)	
Future; median (range), VAS (0 = fear, 10 = confidence)		7.9 (0.5 - 10)	
Body mass index; median (range), kg/m^2^		24.5 (17–48)	
Living together with partner	258		75
Living in rural area	245		72
Private health insurance	139		41
Education			
none or basic	107		31
professional degree	163		48
academic/university degree	67		20
Working situation			
at home	77		23
working	126		37
retired	112		33
other	27		8
Nationality at birth			
Swiss	265		77
EU	50		15
other	27		8
Current treatment			
no	116		34
yes	226		66
endocrine	197		87
chemotherapy	26		12
anti-Her2	13		6
Prior treatment			
surgery	338		99
endocrine	124		36
chemotherapy	189		55
anti-Her2	30		9
radiotherapy	244		71
Concomitant medication for			
diabetes	16		5
hypertension	97		28
depression	24		7
other	54		16

### Physical activity

**Figure 1:**
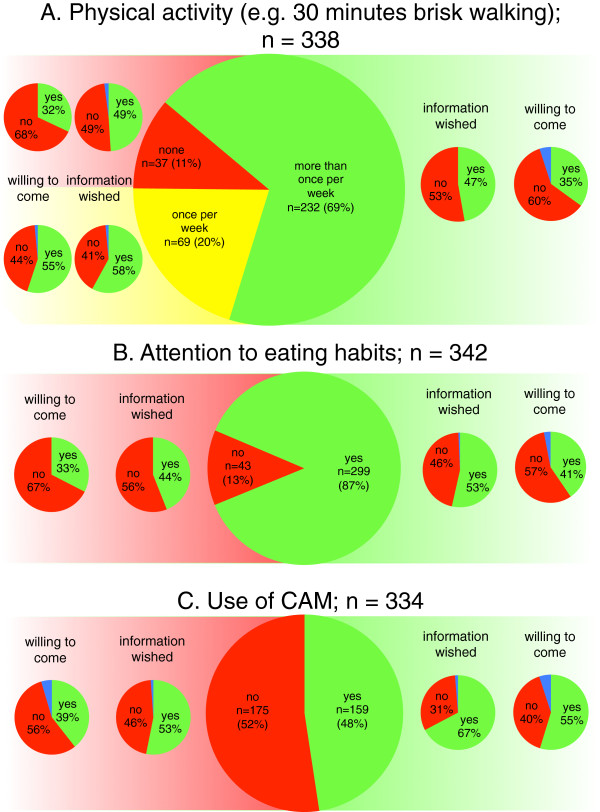
**Current activity, interest in more information and willingness to present for special counselling, total and with respect to current activity in the respective field: A. physical activity; B. attention to eating habits; C. use of complementary and alternative medicine (CAM).** How to read the figure: E.g. in **A**. Physical activity, 20% of 338 patients (n = 69, in yellow) reported to be physically active once per week. Of these 69 patients, 58% (n = 40, in green) indicated the wish to receive more information about the impact of physical activity, whereas 41%, n = 28 (in red), did not wish any specific information about this. The blue segment indicates those patients who did not answer the question (missing data). Of the 69 patients being physically active once per week, 55% (n = 38, in green) indicated to be willing to come for special counselling about physical activity and breast cancer, whereas 44%, n = 30 (in red) did not do so. Figure 1A illustrates that the rate of patients willing to come for counselling is lower than the rate of those wishing more information and that the highest interest in further information and/or activity is indicated by those engaging in some physical activity (i.e. once per week).

More than two thirds (69%) of all patients reported to be physically active more than once per week (i.e. at least twice for ≥ 30 minutes brisk walking or equivalent per week), whereas 20% indicated physical activity once per week (≥ 30 minutes brisk walking or equivalent per week) and only 11% reported not to be physically active (Figure 1A). The rate of interest in further information and willingness to participate in institutional programs (e.g. exercise classes) was highest in the group of patients reporting once weekly physical activity (around 57%). Based on indicated interest in more information or willingness to attend programs, a total of 306 patients were offered a free voucher for a personal instruction by a certified physiotherapist. 140 (46%) of these patients declined the voucher. Of the 166 vouchers which were distributed and after a median follow-up of 31 months (range 16 – 37), only 7 (4%) were utilized for guided training.

In order account for the fact that once weekly PA may not be of great importance, predictive factors for PA were analysed comparing those patients engaging in PA more than once per week with those who were inactive or indicated once weekly PA. Predictive factors for PA were higher age, use of CAM, and less anxiety about the future (Table 2).

**Table 2:** Predictive factors for physical activity, attention to eating habits (diet), CAM, lifestyle change

	**Univariate**				**Multivariate**	
	**OR**	**95% CI**	***P***	**OR**	**95% CI**	***P***
**Physical Activity (more than once per week [n = 232] vs. less [n = 106])**						
Use of CAM	1.8	1.1 - 2.9	0.01	1.8	1.1 - 2.9	0.03
Age (per 10 increase)	1.2	1.0 - 1.5	0.05	1.3	1.0 - 1.6	0.04
Positive about future	1.2	1.0 - 1.3	0.02	1.1	1.0 - 1.3	0.03
Attention to eating	2.6	1.4 - 5.1	< 0.01			
Current treatment	1.5	1.0 - 2.5	0.07			
Private insurance	1.5	1.1 - 2.2	0.02			
Mood (happier on VAS)	1.1	1.0 - 1.3	0.03			
Muscle mass (per 1% increase)	1.1	1.0 - 1.1	< 0.01			
BMI (per 1 kg/m^2^ increase)	0.9	0.9 - 1.0	< 0.01			
Current smoker	0.8	0.6 - 1.0	0.06			
Co-morbidities present	0.7	0.5 - 0.9	< 0.01			
**Special Attention to Diet (yes [n = 299] vs. no [n = 43])**						
Swiss nationality at birth	3.9	1.6 - 9.3	< 0.01	3.7	1.5 - 9.3	< 0.01
Physical activity	2.0	1.3 - 3.0	< 0.01	2.3	1.1 - 4.5	0.02
Higher education	1.5	1.1 - 1.8	< 0.01	1.7	1.0 - 2.7	0.05
Age (per 10 years)	1.4	1.0 - 1.8	0.04			
Use of CAM	2.1	1.1 - 4.1	0.03			
Living with partner	0.4	0.1 - 1.0	0.05			
Her2 positive tumour	0.4	0.2 - 0.9	0.03			
**Use of Complementary and Alternative Medicine (CAM) (yes [n = 159] vs. no [n = 175])**						
Physical activity	1.6	1.2 - 2.3	< 0.01	2.0	1.2 - 3.3	< 0.01
Higher education	1.4	1.1 - 1.6	< 0.01	1.6	1.1 - 2.2	< 0.01
Age (per 10 years)	0.7	0.6 - 0.9	< 0.01	0.7	0.6 - 0.9	< 0.01
Diet	2.1	1.1 - 4.1	0.03			
Comorbidities	0.7	0.5 - 0.9	< 0.01			
Postmenopausal	0.5	0.3 - 0.8	< 0.01			
**Lifestyle Changed after Diagnosis of Breast Cancer (yes [n = 154] vs. no [n = 187])**						
Use of CAM	2.8	1.8 - 4.3	< 0.01	2.2	1.3 - 3.7	< 0.01
Diet	2.0	1.0 - 4.0	0.05	2.2	1.0 - 5.0	0.06
Physical activity	1.4	1.0 - 2.0	0.04	1.6	0.9 - 2.9	0.08
Higher education	1.4	1.2 - 1.7	< 0.01	1.5	1.0 - 2.1	0.03
Mood (happy)	0.9	0.8 - 1.0	0.04	0.9	0.8 - 1.0	0.02
Age (per 10 years increase)	0.5	0.4 - 0.6	< 0.01	0.5	0.4 - 0.7	< 0.01
Postmenopausal	0.5	0.3 - 0.8	< 0.01			
Comorbidities	0.7	0.6 - 0.9	0.01			

### Eating habits (“diet”)

In total, 299 (87%) patients reported paying attention to eating habits. More vegetables/fruit (80%) consumption, “balanced” and (61%), low-fat diet (60%), and organic products (46%) were the most popular (multiple answers were allowed). The rate of patients reporting interest in more information and willingness to come for an addition consultation focussing in such information was similar in these patients paying attention to eating habits compared to those not doing so (Figure 1B).

Predictive factors for paying special attention to eating habits were Swiss nationality at birth, higher education (university degree or university of applied sciences degree), and physical activity (Table 2).

### CAM

Around half of all patients (46%) reported using CAM. Vitamins (38%), teas (29%), homeopathy (19%), herbal medicine (19%), and mistletoe (16%) were the most commonly reported additions to conventional therapies. In most cases (40%), patients had initiated the respective method themselves. A medical doctor or an alternative practitioner was involved in 23% and 14% respectively. Most patients already using CAM were interested in more information on CAM (Figure 1C).

Predictive factors for use of CAM were higher education, physical activity and younger age (Table 2).

### Change of life style with breast cancer diagnosis

154 (45%) of patients indicated having changed their lifestyle in general after breast cancer diagnosis. Of these patients, many reported to live more deliberately (23%) or with less stress/calmer (22%), others to look more after themselves (13%), pay more attention to eating habits (10%), physical activity or to have quit smoking. Overall, several patients reported a positive change of their life, whereas only 4% reported a negative impact (e.g. anxiety).

Predictive factors for a change of lifestyle were use of CAM, paying attention to eating habits, physical activity and these patients tended to have a higher education, be happier and younger (Table 2).

## Discussion

This cross-sectional study of self-reported activities in the fields of physical activity, eating habits, and use of CAM was conducted in a population of patients with early breast cancer (survivors) that may be considered representative for patients with early breast cancer in Eastern Switzerland. Compliance with completion of the questionnaire was high and understanding of the questions was supported by attending specialist breast nurses. Around half of all patients indicated activities, interest in getting more information about the fields and declared willingness to present on an extra occasion. For physical activity, we explored whether patients expressing interest in more activity are really ready to engage actively. All interested patients were offered a free voucher for training with personal instruction by a certified physiotherapist. Interestingly, nearly half of these patients declined this offer despite declaring interest in the questionnaire. Furthermore, only 4% of the vouchers were eventually used. This pattern is not unusual with psychological research reporting that less than half of lifestyle intentions are successfully realised leaving a considerable ‘intention-behaviour gap’ [19]. Besides this, we think that the surprisingly low rate questions the nature of the training offered or the strength of the actual interest and indicated willingness. Many patients who accepted the voucher reported to be physically active already. This may explain in part the low response rate. The rate of declared physical activity was above what had been expected [20] although a direct comparison is limited by the cut off for physical activity used in our study (“at least 60 min/week” and not 150 min/week as recommended to have an impact on disease recurrence [21]). This may also be reflected by a median body mass index of 24.5 kg/m^2^ what is lower than reported in other studies conducted in the field of exercise interventions in Western patients with early breast cancer [20,22]. This possibly reflects a rather healthy life style in many Swiss patients, at least those presenting in our centre. This view is supported by a fairly high life expectancy in Swiss women (84.6 years at birth) and our findings that 87% of all participants reported to pay special attention to eating habits, a rate which is above reported rates in other Western countries such as the United States [23]. Furthermore, a strong predictor for reporting healthy eating was physical activity. Consistent with other data, we did not find a correlation of physical activity or attention to eating habits with participants’ vital signs, body composition, smoking habits or breast cancer characteristics (data not shown) [24]. These findings suggest that activities are rather determined by what is believed to be beneficial by certain patient groups than by the actual risk for recurrence arising from tumour stage and biology.

Nearly half of the participants reported engaging in one or the other field of CAM. Higher education, physical activity and younger age were the strongest predictors of CAM. This rate is similar to that reported in the literature [25,26]. Though most CAM activities reported in our survey can be considered safe, the high rate of complementary therapies suggests that asking patients specifically about the use of such approaches is probably reasonable to ensure safety, especially when combined with conventional therapies. In the case of CAM, there was a remarkable association of the use and willingness to present on another occasion for special counselling, possibly reflecting the strongest health-belief of the fields studied here. It remains uncertain whether such interest may lead to active participation in institutional programs.

Interestingly, nearly all patients indicated to have changed their life after breast cancer diagnosis. Most reported positive effects like finding it easier to look after themselves or to have reduced stress.

The strengths of our study include the prospective design, the use of prospectively defined research questions and hypotheses allowing for robust sample size calculation, the completeness of data and information on non-compliance minimizing selection bias. The studied cohort also underwent external validation by comparison with data obtained from the cancer registry.

Limitations of this questionnaire-based survey include the possibility of answers intended to please (in contrast to entirely anonymous questioning or objective measures) and of inaccurate answers e.g. with self-reported activities where only the options none, once per week or more than once per week were offered. The validity of a 30 minute cut-off can also be questioned especially as 150 minutes of moderate intensity, physical activity is considered the level needed to be associated with a lower recurrence risk [21,27]. Furthermore in some cases the boundary of what is to be considered CAM seems arbitrary (e.g. intake of vitamins or teas) and may not have been interpreted in the same way by all patients.

## Conclusion

A high proportion of breast cancer survivors engage in activities that are believed to have positive effects. Though data supporting this view are largely lacking for CAM with respect to cancer-specific outcomes, implementation of supporting programs are increasingly requested by patient-groups. The content and setting of such programs remain to be elucidated and their impact further evaluated.

## Competing interests

The authors declare that they have no competing interests.

## Authors’ contributions

The study was designed by AT, BT, and TR. Data were collected by AT, BT, MB, MM, SS, MS, and TR. Data were analysed by AT, BT, DD, and TR. The manuscript was written by AT, BT, DD, TR and approved by all authors.

## Pre-publication history

The pre-publication history for this paper can be accessed here:

http://www.biomedcentral.com/1471-2407/13/153/prepub
